# The whole transcriptome and proteome changes in the early stage of myocardial infarction

**DOI:** 10.1038/s41420-019-0152-z

**Published:** 2019-03-04

**Authors:** Yanfei Li, Cuiping Wang, Tingting Li, Linlin Ma, Fangzhou Fan, Yueling Jin, Junwei Shen

**Affiliations:** 1 Shanghai University of Medicine & Health Sciences, Shanghai, 201318 China; 2 Tongji University affiliated Eastern Hospital, Shanghai, 200092 China

## Abstract

As the most severe manifestation of coronary artery disease, myocardial infarction (MI) is a complex and multifactorial pathophysiologic process. However, the pathogenesis that underlies MI remains unclear. Here, we generated a MI mouse model by ligation of the proximal left anterior descending coronary artery. The transcriptome and proteome, at different time points after MI, were detected and analysed. Immune-related pathways, cell cycle-related pathways, and extracellular matrix remodelling-related pathways were significantly increased after MI. Not only innate immune cells but also adaptive immune cells participated in the early stage of MI. Proteins that functioned in blood agglutination, fibrinolysis, secretion, and immunity were significantly changed after MI. Nppa, Serpina3n, and Anxa1, three secreted proteins that can easily be detected in blood, were significantly changed after MI. Our discoveries not only reveal the molecular and cellular changes in MI but also identify potential candidate biomarkers of MI for clinical diagnosis or treatment.

## Introduction

Myocardial infarction (MI), the most severe manifestation of coronary artery disease, causes more than 6.4 million deaths in the US, Europe, and Northern Asia and more than a third of the deaths in developed nations each year^1,2^. Additionally, its medical burden is tremendous; in 2010, more than 1.1 million US hospitalisations were a result of MI, with estimated direct costs of at least US$450 billion, but there were more than 2.4 million patients with MI in 2016^3,4^. Exploration of integrated cellular and molecular characteristics of MI may help to deepen the understanding of myocardial dysfunction during MI, leading to the establishment of personalised treatment and prevention strategies and improvements in patients’ clinical outcomes^4^.

MI is a complex process with different components, including cell hypoxia, apoptosis, migration, fibrosis, and immune cell infiltration. Zhu et al. suggested vascular endothelial cell migration and apoptosis that was induced by iNOS play a crucial role in MI^5^. Ma et al. have demonstrated a dramatic increase in autophagy during the reperfusion phase of cardiac ischaemia^6^. Prabhu et al. and Wang et al. revealed the participation of the inflammatory response in the early stage of MI^7,8^. However, the roles of immune cells in this process have not been identified clearly.

Protein detection plays a major part in medical testing. For example, cardiac troponin is an important diagnostic indicator in MI. Similarly, ANP and BNP are diagnostic markers for heart failure. The changes in protein concentrations during MI progression not only have good clinical research value but also have strong application prospects for clinical diagnosis and treatment. Mass spectrometry (MS) is a key technology of proteomics that is used to identify and quantify proteins and peptides. For example, top-down proteomics reveals that ENH2, which belongs to the PDZ-LIM protein family, is highly expressed in the early stage of MI and contributes to cardiac dysfunction^9^. Proteins implicated in vascular endothelial growth factor (VEGF) signalling and extracellular matrix are significantly up-regulated in the peri-infarct border zone after MI^10^. One recent advance in MS-based targeted proteomics is data-independent acquisition mass spectrometry (DIA-MS)^11^. By characterising different molecules produced from different samples, this method combined with improved bioinformatics analysis is more sensitive in detecting peptides or proteins that are usually missed in traditional MS experiments. The present study is designed to investigate the cellular and molecular changes in different MI stages in a mouse model through the combination of transcriptome and proteome analyses.

## Results

### Functional evaluation of MI mouse model

**Fig. 1:**
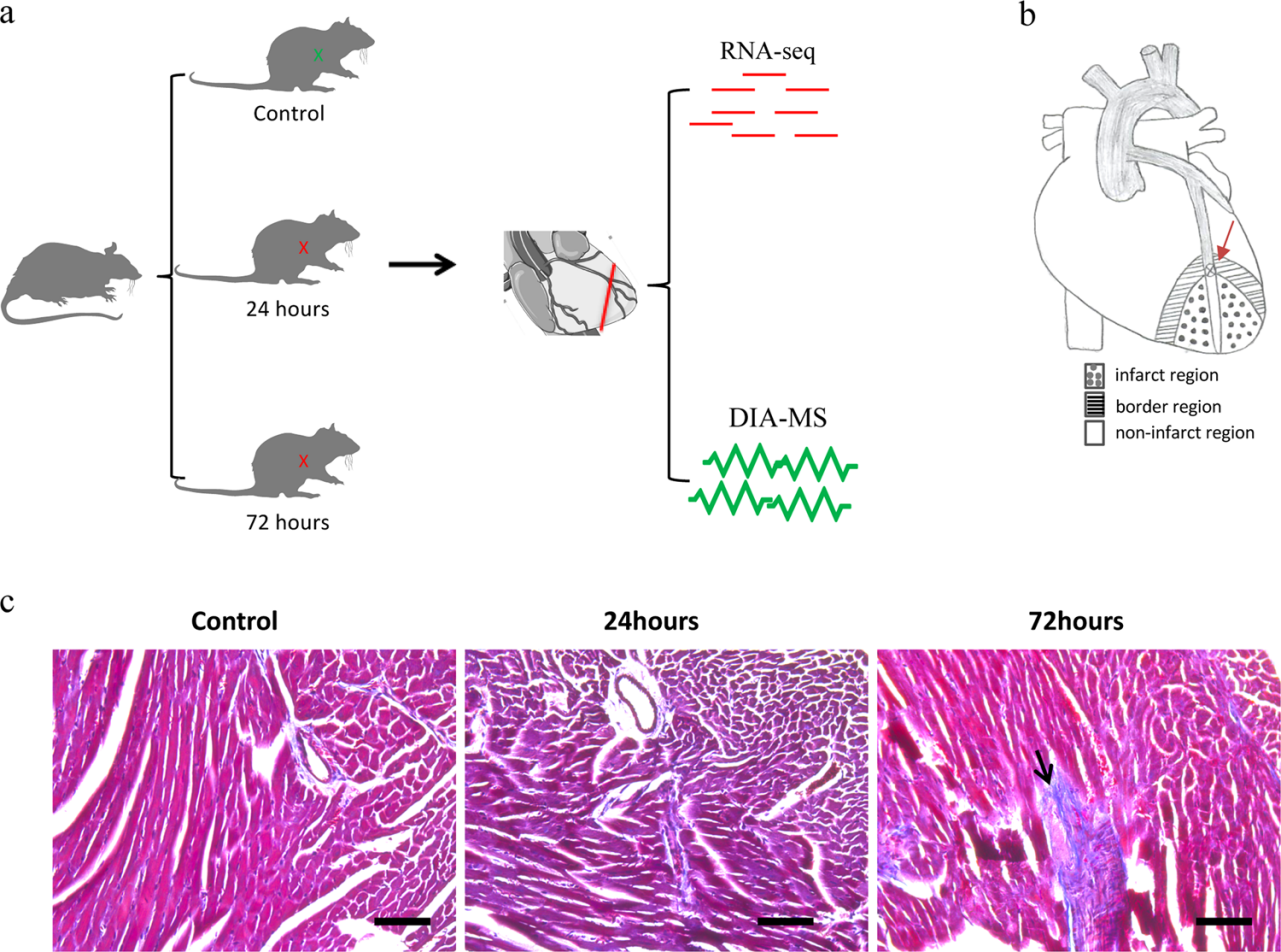
Functional evaluation of myocardial infarction mouse model. **a** Schematic diagram of this study. **b** Schematic diagram of the mouse MI model and infarct tissue. **c** The Masson staining of myocardial tissues. The arrow shows the fibrosis. Bar = 100 μm

To investigate the molecular changes of MI in vivo, C57 mice were treated with MI or sham operation. Then, the infarct left ventricle tissue and control tissue were acquired for RNA-seq and DIA-MS (Fig. 1a). The infarct regions were mainly in the ventricular apical region (Fig. 1b). Myocardial fibrosis is one of the main events after MI, so we evaluated the model by detecting the degree of myocardial fibrosis^8,12^. Masson staining displayed the fibres in the myocardium as blue, and the staining depth reflected the degree of fibrosis. Compared with the control myocardium, fibrotic aggregations were observed in some tissues after 24 h of MI treatment. Additionally, fibrosis was obvious in part of the cardiac tissue in the infarct area after 72 h of MI treatment. These results indicate that this mouse model effectively mimicked the MI process (Fig. 1c).

### Gene expression profiling analysis

**Fig. 2:**
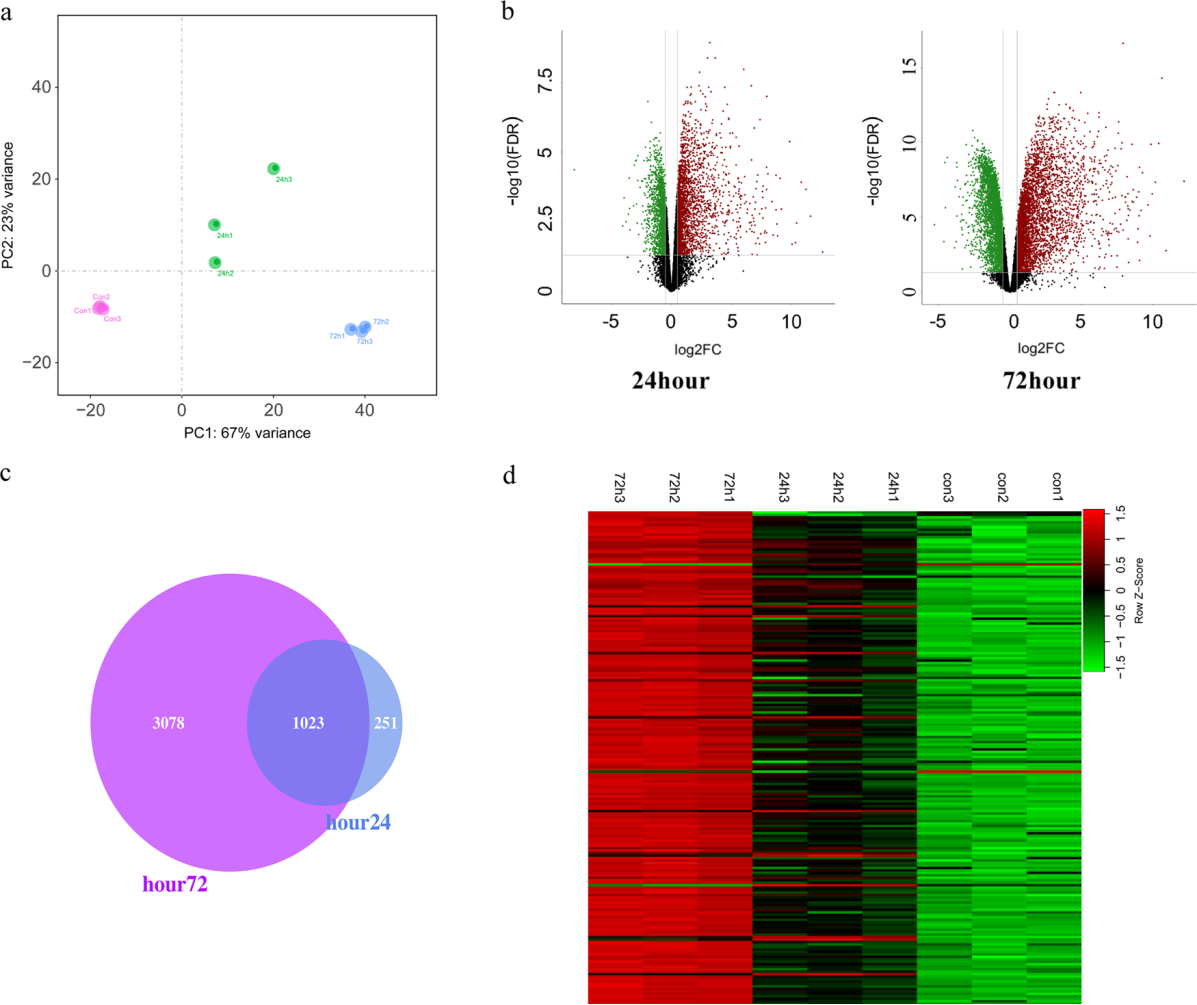
Transcriptome analysis of different time points of MI. **a** Principal component analysis of control and MI groups. **b** Volcano plots of the differentially expressed genes between the control group and MI groups. **c** Venn diagram of differentially expressed genes between the control group and MI group. **d** Heatmap of the top 200 differentially expressed genes between the control group and MI groups

A total of 13,390 genes in the RNA-seq data were detected and analysed. The principal component analysis (PCA) of the RNA-seq data showed that the three groups of samples distinctively clustered into three different categories, indicating the gene expression pattern between these three groups was different (Fig. 2a). Then, we compared the differentially expressed genes (DEGs) and made a volcano plot. Compared with the control group, the gene expression displayed large differences in the 24 h group and the 72 h group (Fig. 2b). The Venn plot showed that the majority (80%) of DEGs between control and 24 h groups were the same as those between control and 72 h groups (Fig. 2c). Finally, the heatmap data showed that the top 200 DEGs could significantly distinguish these three groups of tissues (Fig. 2d).

### Significantly increasing expression of genes involved in immunity and proliferation after MI

**Fig. 3:**
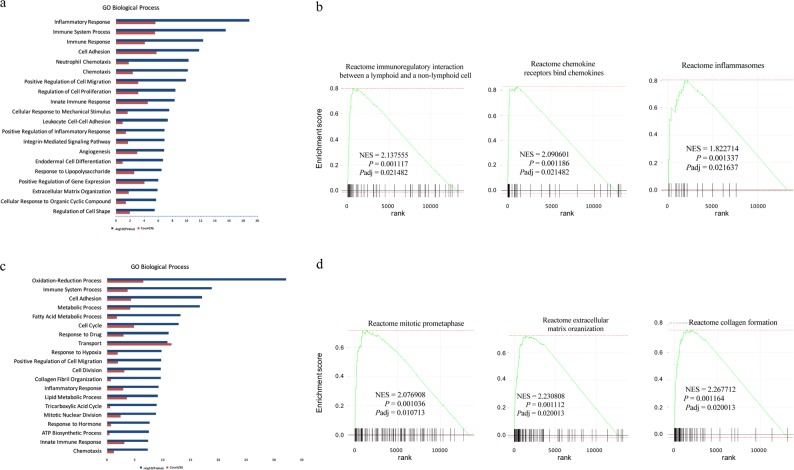
Immunity- and proliferation-related genes are significantly increased after MI. **a** GO biological process analysis of the differentially expressed genes between the control group and the 24 h group. **b** GSEA analysis of transcriptomic gene expression between the control group and the 24 h group. **c** GO biological process analysis of the differentially expressed genes between the control group and the 72 h group. **d** GSEA analysis of transcriptomic gene expression between the control group and the 72 h group. NES normalised enrichment score, *P* nominal *P* value, *P*_adj_ adjusted *P* value

We performed Gene Ontology (GO) analysis of the DEGs between control and 24 h groups. The results showed that more than half of the top 20 categories were associated with immunity, including inflammatory response, immune response, and chemotaxis (Fig. 3a). To further validate this result, we performed GSEA. Consistent with the GO analysis, immune-related pathways also increased significantly. These findings suggest that the immune system was deeply involved in the early stage of MI process (Fig. 3b). Then, we analysed the DEGs between control and 72 h groups and found that, in addition to immunity, cell cycle-related pathways and extracellular matrix remodelling-related pathways were also significantly altered (Fig. 3c, d). The above Masson staining showing partial fibrosis at 72 h agreed with this result.

### Immune cell infiltration during MI

**Fig. 4:**
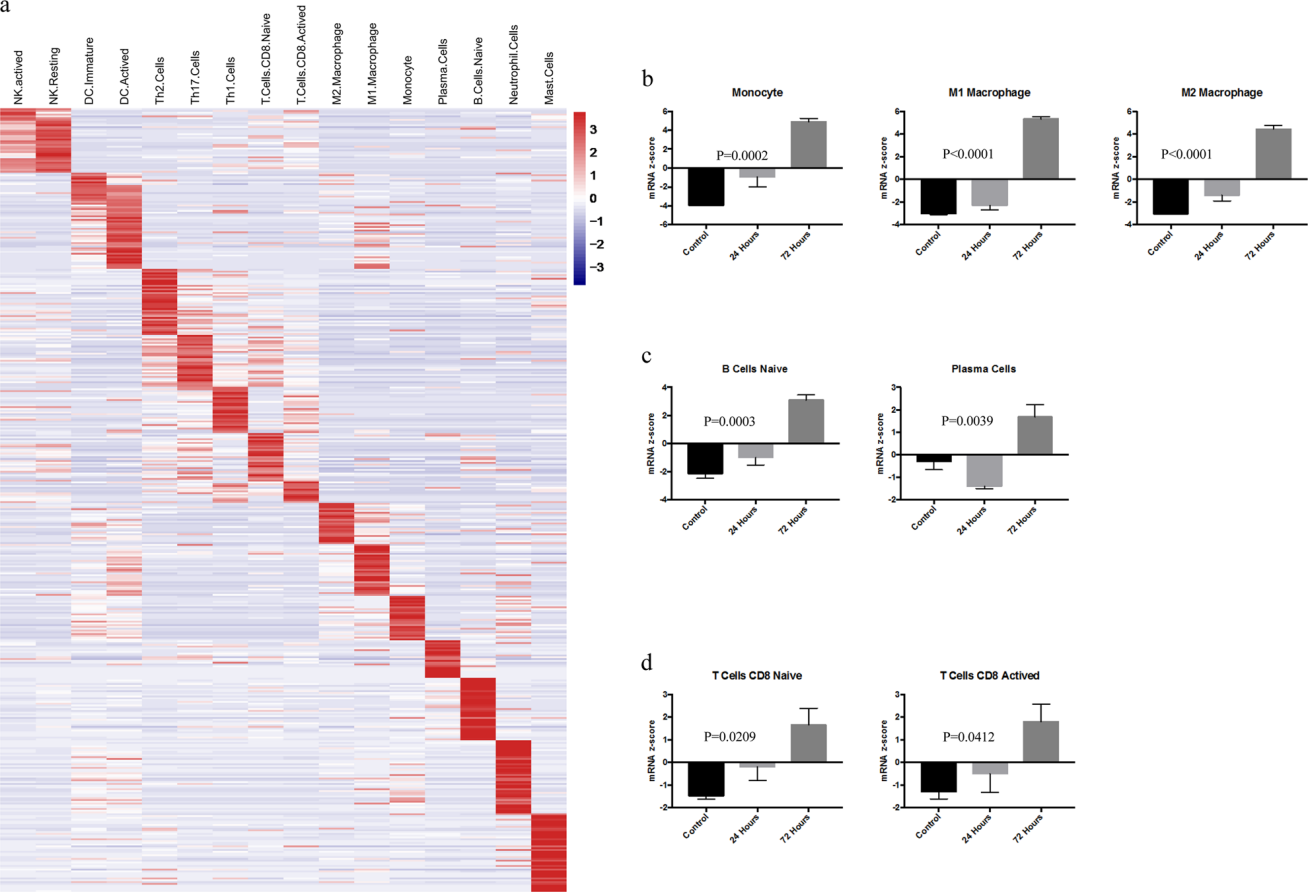
Immune cell infiltration during myocardial infarction. **a** Heatmap of molecular markers specific to different immune cell populations. **b** mRNA *z*-scores of monocytes and macrophages. **c** mRNA *z*-scores of naive and active B cells. **d** mRNA *z*-scores of naive and active T cells

Our data above implicated immunity-associated pathways in the development of MI. Therefore, we focussed on the immune cell infiltration during different MI stages in the following analysis. We selected 16 different immune cell populations for the analysis, and a total of 278 genes were selected as cell-specific markers to define different immune cell signatures according to previous research (Supplementary Table 1). The heatmap showed that these cell-specific markers could distinguish these immune cell populations well (Fig. 4a). Then, we analysed the changes in different immune cell infiltration levels during MI. From the innate immune system, we found that the mRNA *z*-scores of natural killer cells (NK cells), neutrophils cells, and monocytes increased significantly after 24 h of MI compared with the control group (Fig. 4b, Supplemental Fig. 1a and 1c). At 72 h, there was a more significant increase in all kinds of cells, suggesting that immune cells continue to accumulate in the infarct tissue during the development of MI (Fig. 4b, Supplemental Fig. 1a and 1c). We also analysed the changes in adaptive immune cells. Similarly, some kinds of cells were significantly increased in the 24 h group, such as naive B cells and naive CD8^+^ T cells, while in the 72 h group, all kinds of adaptive immune cells were significantly increased compared to the control group (Fig. 4c, d, Supplemental Fig. 1b). These results suggest that the immune cells are deeply and actively involved in the MI process.

### Proteome analysis

**Fig. 5:**
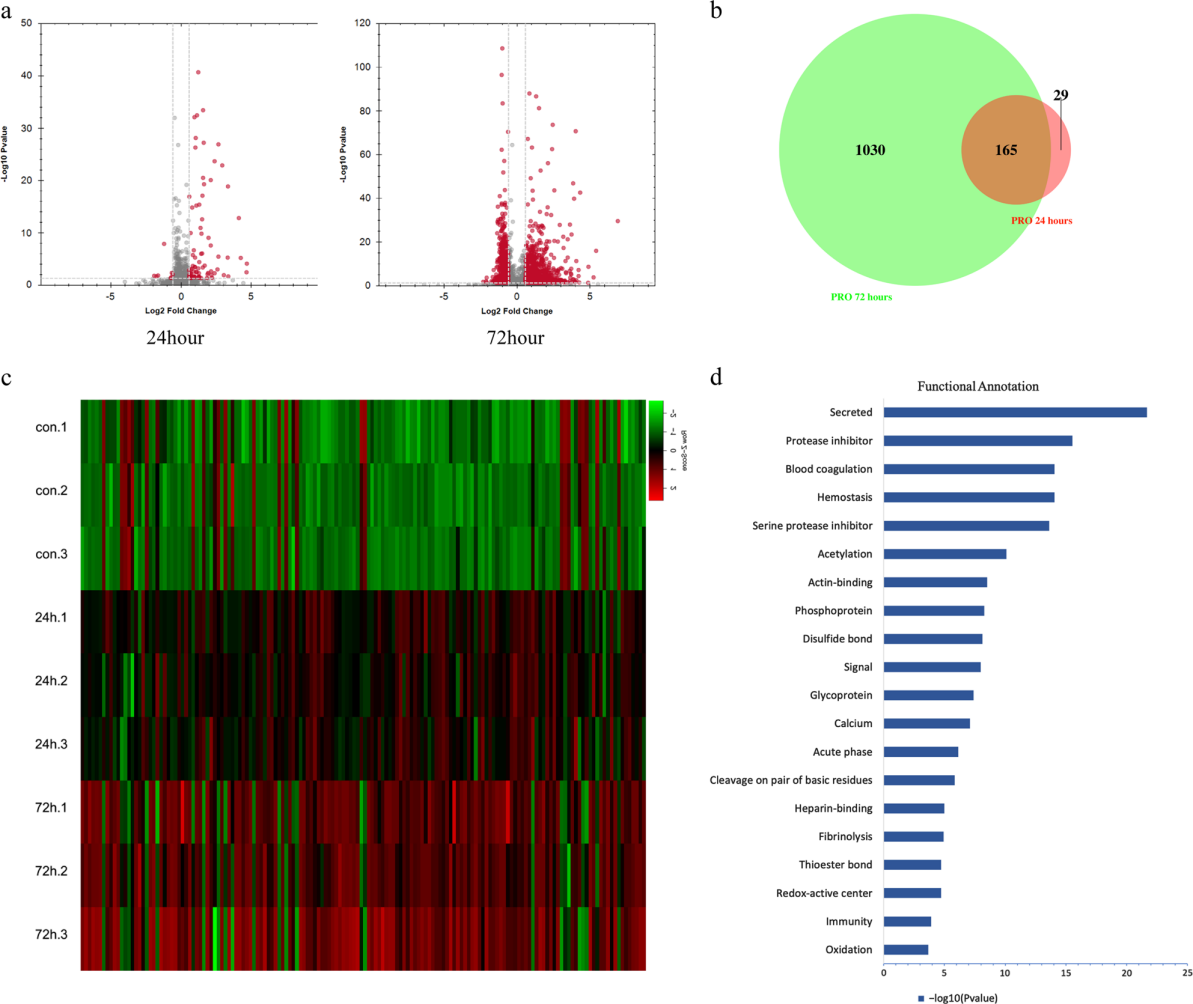
Proteome analysis of different time points of MI. **a** Volcano plots of the differentially expressed proteins between the control group and MI groups. **b** Venn diagram of the differentially expressed proteins between the control group and MI groups. **c** Heatmap of the co-differentially expressed proteins between the control group and both MI groups (24 and 72 h). **d** Functional annotation of the co-differentially expressed proteins between the control group and both MI groups (24 and 72 h) via the DAVID database

We then analysed the tissue of the MI at different times by DIA-MS. The results showed that the expression of certain proteins changed significantly during the development of MI. Volcano plots showed that some protein levels were significantly different between the 24 h group and the control group, and most of them were increased in expression. In the 72 h group, many proteins changed significantly, and most of them were increased in expression as well (Fig. 5a). Most (85%) of the proteins that were differentially expressed in the 24 h group remained significantly different in the 72 h group compared with control (Fig. 5b). These data show that the tissues of the three groups could be well distinguished via these proteins, suggesting a different protein expression pattern in different MI stages (Fig. 5c). We performed functional annotation of these co-variant proteins via the DAVID database (Fig. 5d). The results showed that a portion of them were closely related to immunity. Interestingly, some of these proteins were also associated with fibrinolysis. These results were consistent with the RNA-seq findings above.

### Conjoint analysis of proteome and transcriptome

**Fig. 6:**
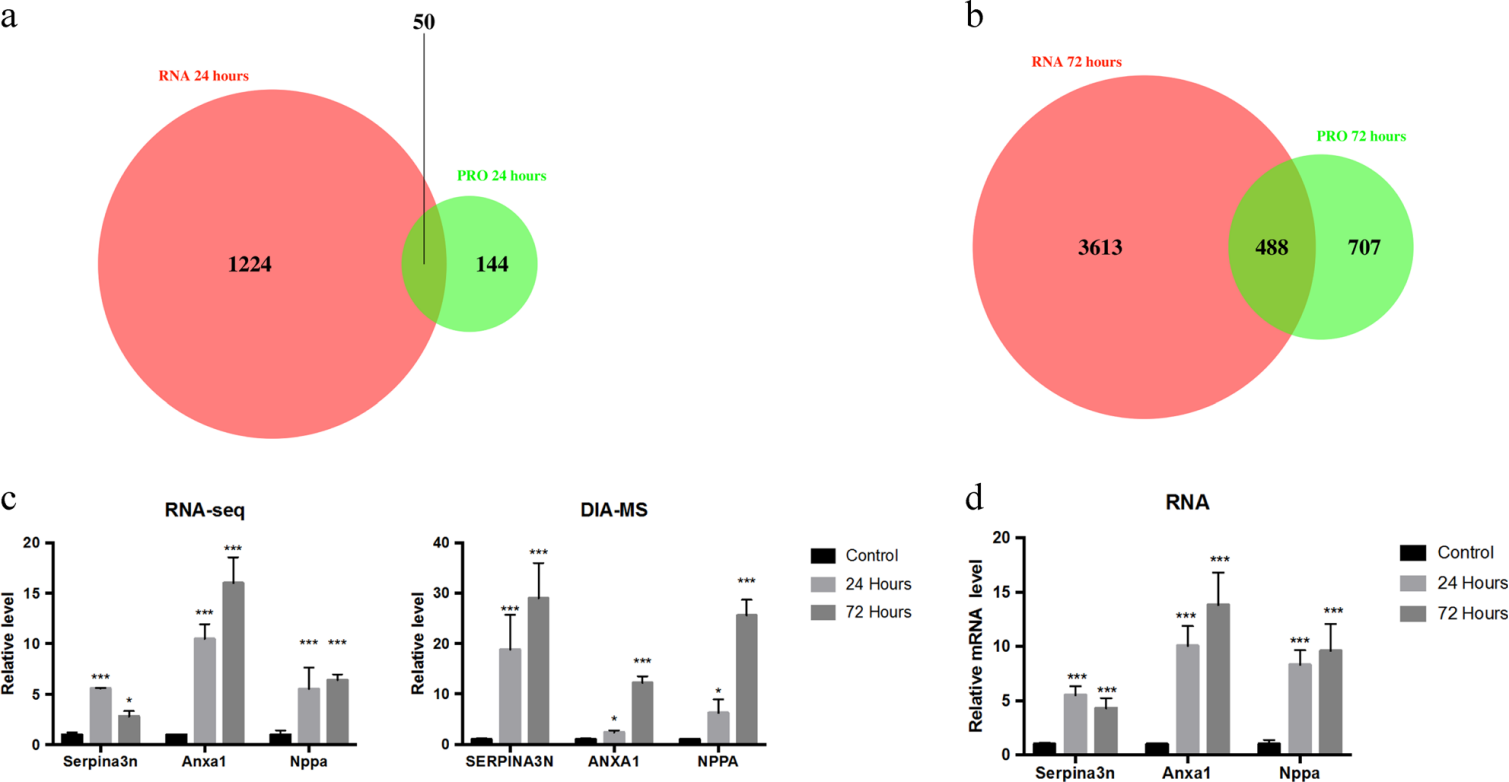
BFLF2 is a nucleocytoplasmic shuttling protein. **a** Venn diagram analysis of the common differential genes in the 24 h group. **b** Venn diagram analysis of the common differential genes in the 72 h group. **c** Differential expression of Serpina3n, Anxa1, and Nppa in the transcriptome and proteome at different time points after MI. **d** qPCR analysis of Serpina3n, Anxa1, and Nppa at different time points after MI (values are mean ± SD, *P < 0.05, two-tailed Student’s *t*-test)

Next, we performed a conjoint analysis of the proteome and transcriptome. Some 25% of the differentially expressed proteins also showed significant changes in the transcriptome profile between control and 24 h groups (Fig. 6a). Approximately 40% of the differentially expressed proteins were also differentially expressed in the transcriptome profile between control and 72 h groups (Fig. 6b). We then analysed the genes and proteins that changed simultaneously in both the proteome and transcriptome data, which may serve as molecular markers of MI (Fig. 6c). Nppa, an important clinical diagnostic indicator of heart failure, significantly increased in the proteome and transcriptome of MI^13^. Serpina3n and Anxa1 also underwent significant changes at various stages during this period. Finally, we validated these results by qPCR (Fig. 6d). The results were consistent with the above analyses. Taken together, our findings suggest that these genes may be potential indicators for MI.

## Discussion

In this study, we detected the gene and protein expression profile in infarct tissues of different stages through transcriptome and proteome data. We found that immune-related genes are one of the most DEG groups after MI. Not only innate immune cells but also adaptive immune cells participated in this process actively, suggesting that the infiltration of immune cells in MI tissues is an important physiopathologic process during MI. There are numerous immune cells in myocardial tissue^14,15^. Though our work confirmed the involvement of immune cells in MI, the biological roles of each immune cell population and the interactions between them need to be identified by further studies. In addition to immune-related genes, genes involved in cell proliferation were also highly differentially expressed in MI tissues. The adult mouse myocardium is mainly composed of cardiomyocytes and fibroblasts. The cardiomyocytes have negligible regenerative capacity. Fibroblasts account for 60% of the total cells, and they could be stimulated to proliferate after MI^16^. We observed that the genes related to fibrosis were significantly up-regulated at 72 h of MI (Fig. 3d). Fibroblasts not only serve as matrix-producing reparative cells but also exhibit a wide range of functions, including inflammatory and immune response^16,17^. These findings, together with our work, suggest that fibroblasts may be major participants in MI.

The pathogenesis of MI is complex and multifactorial. We found that during the early stage of MI, blood agglutination-related proteins underwent significant changes, so they may be involved in the ischaemic cascade of coagulation activation. Immediate interventional treatment is recommended and routinely requires dual antiplatelet therapy, including aspirin and a P2Y12 inhibitor^18^. The fibrinolysis-related pathways were also activated at the protein level, so it seems that the protective mechanism is initiated at an early stage. Interestingly, many protease inhibitors, mainly secreted serine protease inhibitors underwent significant changes. Serine protease inhibitors are closely involved in innate and adaptive immune responses. These results further confirm the important role of the immune system in the early stages of MI. By using DIA-MS, the accuracy and sensitivity of protein and peptide fragment detection are largely improved compared to MS. However, since proteins cannot be amplified like RNA, there is still a gap in the depth and breadth between proteome and transcriptome. Therefore, it is necessary to combine these two methods to investigate the effects of expression changes on cellular and molecular processes.

Through multi-omics analysis, we found that genes such as Serpina3n, Nppa, and Anxa1 had significant changes at both omics levels during MI. Serpina3n, a serine protease inhibitor, is released into the circulation during muscle atrophy^19^. Anxa1, a calcium-dependent phospholipid-binding protein, acts in immune system modulation and cell membrane organisation^20–22^. Nppa is a diagnostic marker for heart failure. Interestingly, all three proteins are secreted proteins and can be detected easily in plasma. Therefore, these proteins are promising biomarkers of MI for diagnosis or treatment. Further clinical investigations are needed to reveal the relationships of these proteins with MI and to screen out valuable biomarkers.

In summary, this study analysed transcriptome and proteome changes at different time points in MI. Immune-related pathways, cell cycle-related pathways, and the collagen formation pathway were significantly changed after MI. We have demonstrated that immune cells participate in the early stage of MI as well. Our findings not only reveal the molecular and cellular changes in MI but also indicate three valuable biomarkers of MI for clinical diagnosis or treatment.

## Materials and methods

### Animals

All mouse studies were performed in accordance with institutional guidelines for the ethical care and use of laboratory animals and were approved by the University Committee on the Care and Use of Laboratory Animals at Shanghai University of Medicine and Health Science (Shanghai, China).

Nine male C57BL/6 mice (8 weeks old) were purchased from Shanghai Slake Experimental Animal Co., Ltd. The mice were divided into three groups, the sham group, the 24 h MI group, and the 72 h MI group. They were anaesthetised by intraperitoneal injection of sodium pentobarbital (50 mg/kg). MI was performed by ligation of the proximal left anterior descending coronary artery. Then, the mice were sacrificed, and the infarcted myocardia was obtained for experiments.

### Quantitative reverse transcription-polymerase chain reaction (qRT-PCR)

Total RNA was isolated using the RNAiso Reagent (TaKaRa, Japan) according to the manufacturer’s instructions. Five hundred nanograms of total RNA was reverse-transcribed using the PrimeScript RT reagent kit (TaKaRa), and resulting cDNAs were analysed by real-time qPCR with iTaq Universal SYBR Green Supermix (Bio-Rad) and SYBR Green qPCR Master Mix (Bio-Rad). Primer sequences are listed in Supplementary Table 1. The fold changes were analysed via the 2^−△△CT^ method.

### Gene expression analysis

Five hundred nanograms of total RNA was used for RNA-seq. Libraries were constructed (NEBNext Ultra RNA Library Prep Kit for Illumina, NEB), and size selection was performed with AMPure XP Beads (Beckman Coulter, Krefeld, Germany). Thirteen PCR cycles were used for library amplification. Amplicons were sequenced on a HiSeq 4000 (150 bp, Illumina, San Diego, USA). RNA-seq data were mapped to the GRCm38.p6 genome.

### Differentially expressed gene analysis

The DEGs between control, 24-h, and 72-h groups were analysed by the edgeR R/Bioconductor package^23^. Genes with log_2_|FC| ≥ 1 and *P* < 0.01 were considered to be significant DEGs. The DEG data are listed in Supplementary Table 1. GO analysis of DEGs was performed via The Database for Annotation, Visualization and Integrated Discovery (DAVID) (https://david.ncifcrf.gov/)^24^.

### Gene set enrichment analysis

Fast Gene Set Enrichment Analysis (fGSEA) was performed via the fgsea R/Bioconductor package. The log_2_(FC) value of each genes calculated by the edgeR package was used as the ranking metric input. We used the REACTOME pathways in the C2 collection in the Molecular Signatures Database (MSigDB) as the gene sets in the analysis. This sub-collection contained 674 gene sets, which were annotated pathways from the REACTOME database. The gene sets contained in the MSigDB were from a wide variety of sources and related to a variety of species, mostly human. Therefore, we downloaded the Mouse version of the MSigDB, which mapped all gene sets to mouse orthologues, from http://bioinf.wehi.edu.au/software/MSigDB/index.html.

### Immune cell population analysis

Various immune cell signatures containing genes specific to these cells can define different immune cell phenotypes (Supplementary Table 1)^25^. The mRNA *z*-scores of immune cell signatures were calculated through the use of normalised RNA sequencing data via the function scale in R to evaluate the immune cell infiltration in myocardial tissue^26^.

### DIA-MS

Nine myocardium samples were lysed with lysis buffer (1% SDS, 7 M urea, 1× Protease Inhibitor Cocktail (Roche Ltd. Basel, Switzerland)) and centrifuged at 15,000 rpm for 15 min at 4 °C. The supernatant was collected, and the protein concentration of the supernatant was tested by using the BCA protein assay (Beyotime Ltd. Shanghai, China). The protein samples were adjusted to 100 μl with 8 M urea. Then, 2 μl 0.5 M TCEP was added, and the samples were incubated at 37 °C for 1 h. Next, 4 μl 1 M iodoacetamide was added to each sample, followed by incubation for 40 min in the dark at room temperature. After that, five volumes of −20 °C pre-chilled acetone was added to precipitate the proteins overnight at −20 °C. The precipitates were washed by 1 ml pre-chilled 90% acetone aqueous solution twice and then re-dissolved in 100 μl 100 mM TEAB. Sequence-grade modified trypsin (Promega, WI, USA) was added at the ratio of 1:50 (enzyme:protein, weight:weight) to digest the proteins at 37 °C overnight. The peptide mixtures were desalted by a C18 ZipTip, quantified by the PierceTM Quantitative Colorimetric Peptide Assay (23275) and then lyophilised by a SpeedVac.

The lyophilised peptide mixtures were re-dissolved in buffer A (20 mM ammonium formate, pH 10.0, adjusted with ammonium hydroxide), then fractionated by high-pH separation using an Ultimate 3000 system (Thermo Fisher Scientific, MA, USA) connected to a reverse-phase column (XBridge C18 column), 4.6 mm × 250 mm, 5 μm, (Waters Corporation, MA, USA). High-pH separation was performed with a linear gradient, starting from 5% B to 45% B in 40 min (B: 20 mM ammonium formate in 80% ACN, pH 10.0, adjusted with ammonium hydroxide). The column was re-equilibrated at the initial condition for 15 min. The column flow rate was maintained at 1 ml/min, and the column temperature was maintained at 30 °C. Ten fractions were collected and each fraction was dried in a vacuum concentrator for the next step.

Then the peptides were re-dissolved in 30 μl solvent A (0.1% formic acid in water) and analysed by on-line nanospray LC-MS/MS on an Orbitrap Fusion Lumos (Thermo Fisher Scientific, MA, USA) coupled to a Nano ACQUITY UPLC system (Waters Corporation, MA, USA). 10 μl of peptides were loaded onto the trap column (Thermo Fisher Scientific) with a flow rate of 300 nl/min and subsequently separated on the analytical column (Acclaim PepMap C18, 75 μm × 15 cm) with a nonlinearly increased gradient from 3% B (0.1% formic acid in ACN) to 7% B in the first 3 min, from 7% B to 20% B in minutes 3–83, from 20% B to 32% B in minutes 83–107, and from 32% B to 90% B in minutes 107–108, followed by holding at 90% B for 12 min. The column temperature was 40 °C. An electrospray voltage of 2.1 kV versus the inlet of the mass spectrometer was used. The mass spectrometer was run under data-independent acquisition mode and automatically switched between MS and MS/MS mode.

## Supplementary information

Supplemental Figure legend

Supplementary Figure 1

Supplementary Table 1
