# Supplementary material for: The whole transcriptome and proteome changes in the early stage of myocardial infarction
Source: Cell Death Discov. 2019 Mar 4;5:73. doi: 10.1038/s41420-019-0152-z (PMC6399251; doi:10.1038/s41420-019-0152-z)
Supplement: Supplementary file 1 — Supplemental Figure legend [file 41420_2019_152_MOESM1_ESM.docx]

Supplemental Fig. 1. Immune cell infiltration during myocardial infarction.

(a) mRNA z-scores of neutrophils and mast cells.

(b) mRNA z-scores of T-helper cells.

(c) mRNA z-scores of naïve and active NK cells.
