# Supplementary figures and images for: The whole transcriptome and proteome changes in the early stage of myocardial infarction
Source: Cell Death Discov. 2019 Mar 4;5:73. doi: 10.1038/s41420-019-0152-z (PMC6399251; doi:10.1038/s41420-019-0152-z)

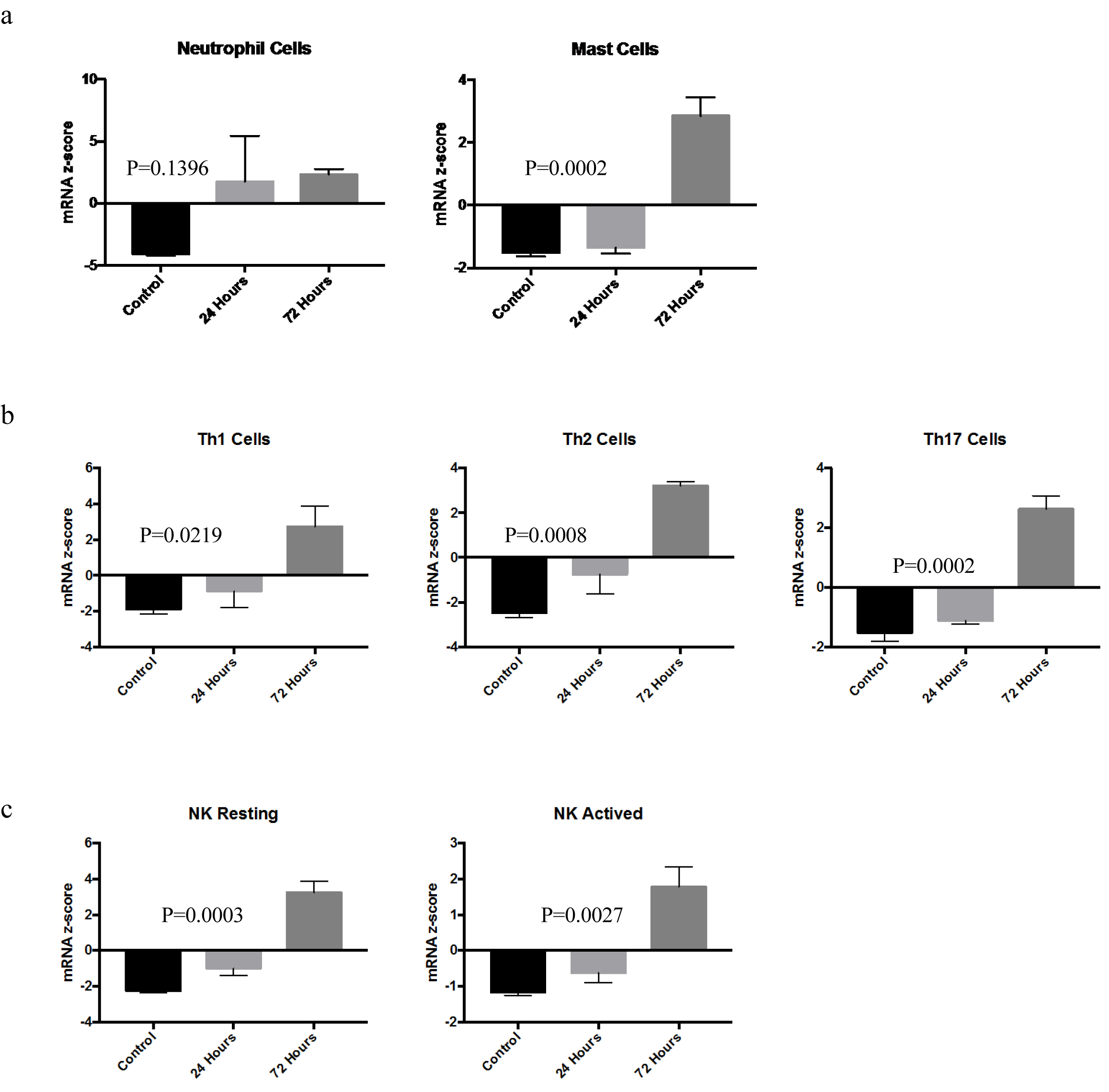

Supplement: Supplementary file 2 — Supplementary Figure 1 [file 41420_2019_152_MOESM2_ESM.tif]
